# Impact of oral vitamin D supplementation on serum 25-hydroxyvitamin D levels in oncology

**DOI:** 10.1186/1475-2891-9-60

**Published:** 2010-11-23

**Authors:** Pankaj G Vashi, Kristen Trukova, Carolyn A Lammersfeld, Donald P Braun, Digant Gupta

**Affiliations:** 1 Cancer Treatment Centers of America® (CTCA) at Midwestern Regional Medical Center, 2610 Sheridan Road, Zion, IL, 60099, USA

## Abstract

**Background:**

Serum 25-hydroxyvitamin D [25(OH)D] is the major circulating form of vitamin D and a standard indicator of vitamin D status. Emerging evidence in the literature suggests a high prevalence of suboptimal vitamin D (as defined by serum 25(OH)D levels of <32 ng/ml) as well as an association between lower serum levels and higher mortality in cancer. We investigated the effect of oral vitamin D supplementation as a means for restoring suboptimal levels to optimal levels in cancer.

**Methods:**

This is a retrospective observational study of 2198 cancer patients who had a baseline test prior to initiation of cancer therapy at our hospital to evaluate serum 25(OH)D levels between Jan 08 and Dec 09 as part of their initial nutritional evaluation. Patients with baseline levels of < = 32 ng/ml (n = 1651) were considered to have suboptimal serum 25(OH)D levels and were supplemented with 8000 IU of Vitamin D3 (four 2000 IU D3 capsules) daily as part of their nutritional care plan. The patients were retested at their first follow-up visit. Of 1651 patients, 799 were available for follow up assessment. The mean serum 25(OH)D levels were compared in these 799 patients across the 2 time points (baseline and first follow-up) using paired sample t-test. We also investigated the factors associated with response to vitamin D supplementation.

**Results:**

Of 2198 patients, 814 were males and 1384 females. 1051 were newly diagnosed and treated at our hospital while 1147 were diagnosed and treated elsewhere. The mean age at presentation was 55.4 years. The most common cancer types were breast (500, 22.7%), lung (328, 14.9%), pancreas (214, 9.7%), colorectal (204, 9.3%) and prostate (185, 8.4%). The mean time duration between baseline and first follow-up assessment was 14.7 weeks (median 10.9 weeks and range 4 weeks to 97.1 weeks). The mean serum 25(OH)D levels were 19.1 ng/ml (SD = 7.5) and 36.2 ng/ml (SD = 17.1) at baseline and first follow-up respectively; p < 0.001. Patients with prostate and lung cancer had the highest percentage of responders (70% and 69.2% respectively) while those with colorectal and pancreas had the lowest (46.7% each). Similarly, patients with serum levels 20-32 ng/ml at baseline were most likely to attain levels > 32 ng/ml compared to patients with baseline levels < 20 ng/ml.

**Conclusions:**

The response to supplementation from suboptimal to optimal levels was greatest in patients with prostate and lung cancer as well as those with baseline levels between 20-32 ng/ml. Characteristics of non-responders as well as those who take longer to respond to supplementation need to be further studied and defined. Additionally, the impact of improved serum 25(OH)D levels on patient survival and quality of life needs to be investigated.

## Background

A blood calcidiol [25(OH)D] level is the most accepted way to determine vitamin D status. The appropriate thresholds to define vitamin D deficiency are debated with some investigators considering levels < = 32 ng/mL as "deficient" while others consider this level to be "suboptimal" [1,2]. The most widely accepted optimal level of serum 25(OH)D is 35-55 ng/mL [1]. For cancer prevention, the desirable 25(OH)D levels have been shown to be 36-48 ng/mL [3].

Hypovitaminosis D has been found to be associated with a variety of cancers including prostate [4,5], multiple myeloma, colorectal and breast [6]. Some studies have shown 25(OH)D levels to have an inverse relation with cancer mortality [7-10] while others consider suboptimal levels as a potential risk factor [11]. A study demonstrated that geographic variation in cancer mortality rates in the US is associated with variations in solar ultraviolet-B radiation exposure [12]. The evidence that higher 25(OH)D levels through increased sunlight exposure or dietary supplement intake inhibit colorectal carcinogenesis is substantial [13,14]. Biologic evidence for an association between 25(OH)D and risk for prostate cancer is also reported but the epidemiologic data have not been definitive [15]. Nevertheless, the available clinical data suggest that vitamin D influences cancer prevalence, risk and survival. This provides a sound rationale for studies that assess 25(OH)D levels in cancer patients.

Several studies have addressed the impact of vitamin D supplementation on serum levels of circulating 25(OH)D in healthy adults [16-19], children [20], older population, [21,22] lactating women [23], as well as in patients with rheumatic disease [24], chronic kidney disease [25], hip fracture [26] and hypovitaminosis [27]. Surprisingly, however, data on the impact of vitamin D supplementation on circulating 25(OH)D levels in patients with cancer has not been investigated adequately and many unanswered questions remain [28-30]. The current study has attempted to address the issue of the effectiveness of vitamin D supplementation as a means for restoring suboptimal levels to optimal levels utilizing an oral vitamin D formulation in a large heterogeneous population of patients with cancer.

## Methods

### Study Sample

A retrospective observational study performed on a case series of 2198 heterogeneous cancer patients treated at Cancer Treatment Centers of America^® ^(CTCA) at Midwestern Regional Medical Center (MRMC) between January 2008 and December 2009. Only patients with a histologically confirmed diagnosis of cancer were included in this study. This study examined the serum 25(OH)D levels of all new patients presenting to our institution as part of a routine nutritional status screening prior to initiation of anticancer therapy. The study did not restrict patients with respect to treatment history, tumor histology or stage. The largest cohorts in the study had a diagnosis of either breast, colorectal, lung, prostate or pancreatic cancer. This study was approved by the Institutional Review Board at Midwestern Regional Medical Center (MRMC).

### Vitamin D Assessment and Supplementation

All patients underwent a baseline serum 25(OH)D assessment before undergoing any treatment at our hospital. Serum was collected at the MRMC laboratory, packed in coolpacks and sent to the Laboratory Corporation of America (Raleigh, NC) where a chemiluminescence immune assay (CLIA, DiaSorin Liasion assay) was used to measure 25(OH)D. Serum samples were incubated with antivitamin-D coated microparticles and isoluminol derivative-conjugated 25(OH)D before measurement of chemiluminescent signals. Analysis was completed within 48 hours of collection. The DiaSorin Liasion 25(OH)D assay has been clinically validated to be comparable in accuracy and precision to the radioimmunoassay (RIA). This method uses the same particles used in the DiaSorin RIA technique. Studies have found this to be a rapid, accurate, and precise tool for the measurement of serum 25(OH)D [31,32].

Patients with baseline levels of < = 32 ng/ml (n = 1651) were considered to have suboptimal serum 25(OH)D levels and were supplemented with 8000 IU of vitamin D3 (two 2000 IU D3 capsules twice daily with food) as part of their nutritional care plan [1,33,34]. In our study, patients were encouraged to use a capsule form of vitamin D3 which was made available to all patients through our pharmacy; however, tablet or liquid formulations were not prohibited. Patients with hyperglycemia, history of kidney stones, parathyroid disease, sarcoidosis and hypercalcemia were not supplemented. Patients were encouraged to take vitamin D with food to increase absorption. The patients were retested at their first follow-up visit. Responders were defined as those whose 25(OH)D levels reached > 32 ng/mL after 8 weeks of supplementation while non-responders were those whose 25(OH)D levels remained < = 32 ng/mL after 8 weeks of supplementation. Supplementation was continued at 8000 IU/day for another 8 weeks for non-responders while responders were put on a maintenance dose of 2000 IU/day. Non-responders were also questioned about compliance, which was reinforced.

### Data Analysis and Statistical Methods

The following covariates were evaluated: type of tumor, age at presentation, gender, stage at diagnosis and prior treatment history. Associations between baseline 25(OH)D levels and covariates were assessed using chi-square test, 2 sample t-test, ANOVA, correlation analysis and simple linear regression depending upon the underlying distribution of the variables evaluated. Vitamin D was used as both continuous as well as dichotomous variable (< = 32 ng/ml and > 32 ng/ml). Differences in 25(OH)D levels at baseline and after treatment with high dose vitamin D supplements were assessed using paired sample t-test. The distribution of differences in 25(OH)D levels were examined in relation to the above covariates using chi-square test, 2 sample t-test, ANOVA, correlation analysis and simple linear regression depending upon the underlying distribution of the variables evaluated. All data were analyzed using SPSS 17.0 (SPSS Inc., Chicago, IL, USA). All analyses were two-tailed, and p values were considered significant when < 0.05.

## Results

### Vitamin D at Baseline

2198 patients were tested between January 2008 and December 2009 at MRMC. Table 1 describes the baseline characteristics of our patient population stratified by serum 25(OH)D levels using a cut-off of 32 ng/ml. Of 2198 patients, 1651 (75.1%) were suboptimal in baseline serum 25(OH)D (with levels < = 32 ng/ml). Over 70% of both new and previously treated patients were suboptimal suggesting that the prevalence of suboptimal levels is not related to treatment history. Colorectal cancer patients had the highest prevalence of suboptimal vitamin D (85.3%) while breast cancer patients had the lowest (69.4%). Table 2 compares the mean values of baseline serum 25(OH)D across different types of cancers. Consistent with the results reported in Table 1, patients with breast cancer were found to have the highest average 25(OH)D levels of 27.9 ng/ml whereas those with colorectal cancer were found to have the lowest average of 21.6 ng/ml. When comparing the mean values of baseline serum 25(OH)D across gender, prior treatment history and stage at diagnosis, we found no statistically significant relationship between any of these covariates and serum 25(OH)D levels. Finally, there was no correlation between age at presentation and baseline 25(OH)D levels (Pearson r = 0.014, p = 0.52).

**Table 1:** Baseline patient characteristics (N = 2198)

Characteristic	Serum 25(OH)D levels		p-value
		
	< = 32 ng/ml (n = 1651)	> 32 ng/ml (n = 547)	
Gender			
Males	628 (77.1)	186 (22.9)	Chi-square
Females	1023 (73.9)	361 (26.1)	0.09

Age at Presentation			
Mean	55.2	55.7	2 sample t-test
Median	55.9	55.8	0.32
Range	18.6 - 92.1	21.1 - 89.6	

Treatment history			
Newly Diagnosed	769 (73.2)	282 (26.8)	Chi-square
Previously Treated	882 (76.9)	265 (23.1)	**0.04**

Cancer Site			
Breast	347 (69.4)	153 (30.6)	
Lung	268 (81.7)	60 (18.3)	
Pancreas	157 (73.4)	57 (26.6)	Chi-square
Colorectal	174 (85.3)	30 (14.7)	**< 0.001**
Prostate	132 (71.4)	53 (28.6)	
Others	573 (74.7)	194 (25.3)	

Stage at Diagnosis			
Stage 0	21 (75)	7 (25)	
Stage 1	194 (73.2)	71 (26.8)	
Stage 2	332 (73.8)	118 (26.2)	Chi-square
Stage 3	394 (76.7)	120 (23.3)	0.76
Stage 4	566 (74.8)	191 (25.2)	
Indeterminate	144 (78.3)	40 (21.7)	

SD = Standard Deviation; Numbers in parenthesis for cancer site, gender, treatment history and stage at diagnosis are row percentages

**Table 2:** Mean baseline serum 25(OH)D levels stratified by top 5 cancer sites (N = 2198)

Site of tumor	Mean 25(OH)D in ng/mL	SD	ANOVA p-value
All Cancers (n = 2198)	25.1	15.1	**< 0.001**
	
Breast (n = 500)	27.9	17.6	
	
Lung (n = 328)	22.7	11.9	
	
Pancreas (n = 214)	24.4	15.3	
	
Colorectal (n = 204)	21.6	11.4	
	
Prostate (n = 185)	26.8	14.6	
	
Others (n = 767)	25.0	15.0	

SD = Standard Deviation

### Vitamin D at First Follow-up

Of 1651 patients who had suboptimal serum 25(OH)D levels at baseline, 799 were available for follow up assessment providing a follow-up rate of 48.4%. Patients available for first follow-up (n = 799) differed from those not available (n = 852) with regard to several baseline characteristics such as cancer type, stage, gender and treatment history as reported in Table 3. The mean time duration between baseline and first follow-up assessment was 14.7 weeks (median 10.9 weeks and range 4 weeks to 97.1 weeks). There were no episodes of intolerance to vitamin D and no toxicity was reported or observed in any patient. Of 799 patients, 441 (55.2%) were responders and 358 (44.8%) were non-responders at first follow-up. Supplementation was continued at 8000 IU/day for another 8 weeks for non-responders while responders were put on a maintenance dose of 2000 IU/day. The results for second follow-up after 8 weeks are not available yet and therefore not included in the present analysis. Table 4 compares the mean scores of 25(OH)D at baseline and first follow-up in these 799 patients for all cancers combined as well as stratified by the top 5 cancer sites. The difference in means across the two time points was statistically significant in the total sample as well as within each stratum of cancer type. The highest improvement in mean serum levels was observed in lung cancer patients (22.7) while lowest improvement was seen in colorectal cancer patients (13.2), the difference being statistically significant.

**Table 3:** Comparison of baseline characteristics of patients available for follow-up versus those not available

Characteristic	Patients with follow-up data (N = 799)	Patients without follow-up data (N = 852)	p-value
Mean age at presentation (SD)	55.0 (9.8)	55.5 (10.1)	2 sample t-test 0.37

Cancer Site			
Breast	182 (52.4)	165 (47.6)	
Lung	120 (44.8)	148 (55.2)	
Pancreas	60 (38.2)	97 (61.8)	Chi-square
Colorectal	90 (51.7)	84 (48.3)	**0.03**
Prostate	60 (45.5)	72 (54.5)	
Others	287 (50.1)	286 (49.9)	

Gender			
Males	266 (42.4)	362 (57.6)	Chi-square
Females	533 (52.1)	490 (47.9)	**< 0.001**

Treatment History			
Newly Diagnosed	410 (53.3)	359 (46.7)	Chi-square
Previously Treated	389 (44.1)	493 (55.9)	**< 0.001**

Stage at Diagnosis			
Stage 0	8 (38.1)	13 (61.9)	
Stage 1	106 (54.6)	88 (45.4)	
Stage 2	164 (49.4)	168 (50.6)	Chi-square
Stage 3	208 (52.8)	186 (47.2)	**0.02**
Stage 4	244 (43.1)	322 (56.9)	
Indeterminate	69 (47.9)	75 (52.1)	

SD = Standard Deviation

Numbers in parenthesis for cancer site, gender, treatment history and stage at diagnosis are row percentages

**Table 4:** Comparison of mean serum 25(OH)D levels at baseline and first follow-up stratified by top 5 cancer sites (N = 799)

Site of tumor	Baseline Mean 25(OH)D in ng/mL (SD)	Follow-up Mean 25(OH)D in ng/mL (SD)	Paired t-test p-value
All Cancers (n = 799)	19.1 (7.5)	36.2 (17.1)	**< 0.001**

Breast (n = 182)	19.7 (8.0)	37.6 (16.8)	**< 0.001**

Lung (n = 120)	18.4 (7.3)	41.1 (18.9)	**< 0.001**

Pancreas (n = 60)	16.2 (7.7)	30.8 (14.2)	**< 0.001**

Colorectal (n = 90)	18.7 (7.7)	31.9 (16.2)	**< 0.001**

Prostate (n = 60)	20.2 (6.9)	42.1 (15.9)	**< 0.001**

Others (n = 287)	19.5 (7.1)	34.6 (16.8)	**< 0.001**

SD = Standard Deviation

### Factors Associated with Response to Supplementation

Table 5 compares responders versus non-responders with regard to cancer type, gender, prior treatment history, stage at diagnosis and baseline 25(OH)D levels. Patients with prostate and lung cancer had the highest percentage of responders (70% and 69.2% respectively) while those with colorectal and pancreas had the lowest (46.7% each). The distribution of responders versus non-responders did not differ by gender, treatment history and stage at diagnosis. Age at presentation was positively and weakly correlated with improvement in 25(OH)D levels (Pearson r = 0.08, p = 0.02). Interestingly, baseline 25(OH)D levels were a strong predictor of improvement in serum levels such that lower values at baseline were correlated with a higher improvement (Pearson r = -0.20, p < 0.001). Upon simple linear regression analysis, every 1 ng/mL decrease in baseline vitamin D level was associated with an improvement of 0.44 ng/mL (95% CI: 0.29 - 0.60 ng/mL) at first follow-up. However, when we compared the distribution of responders across three categories of baseline 25(OH)D levels, we found that patients with serum levels 20-32 ng/ml at baseline were most likely to attain levels > 32 ng/ml compared to patients with baseline levels < 20 ng/ml.

**Table 5:** Comparison of responders versus non-responders with regard to cancer type, gender, prior treatment history, stage at diagnosis and baseline 25(OH)D levels (N = 799)

Characteristic	Responders (n = 441)	Non-responders (n = 358)	Chi-square p-value
Cancer Site			
Breast	103 (56.6)	79 (43.4)	
Lung	83 (69.2)	37 (30.8)	
Pancreas	28 (46.7)	32 (53.3)	**< 0.001**
Colorectal	42 (46.7)	48 (53.3)	
Prostate	42 (70)	18 (30)	
Others	143 (49.8)	144 (50.2)	

Gender			
Males	153 (57.5)	113 (42.5)	0.35
Females	288 (54.0)	245 (46.0)	

Treatment History			
Newly Diagnosed	233 (56.8)	177 (43.2)	0.34
Previously Treated	208 (53.5)	181 (46.5)	

Stage at Diagnosis			
Stage 0	3 (37.5)	5 (62.5)	
Stage 1	56 (52.8)	50 (47.2)	
Stage 2	91 (55.5)	73 (44.5)	0.82
Stage 3	117 (56.3)	91 (43.8)	
Stage 4	139 (57)	105 (43)	
Indeterminate	35 (55.2)	34 (44.8)	

Baseline serum 25(OH)D			
< 12 ng/ml	55 (35.3)	101 (64.7)	
12-20 ng/ml	145 (55.1)	118 (44.9)	**< 0.001**
20-32 ng/ml	241 (63.4)	139 (36.6)	

Numbers in parenthesis are row percentages

## Discussion

Vitamin D has been hypothesized to have an association with cancer risk and survival, and several studies in the literature document a high prevalence of vitamin D deficiency in cancer patients [35-39]. Although vitamin D deficiency is now more recognized in the oncology population, relatively less is known about patients' response to supplementation, dosing regimens, and overall benefits other than bone health. The current study was undertaken to investigate the effectiveness of oral vitamin D supplementation to restore suboptimal serum 25(OH)D levels in a large heterogeneous population of patients with cancer.

The response to supplementation (in terms of restoration from suboptimal levels to optimal levels) was most pronounced in patients with lung and prostate cancer and least in those with colorectal and pancreatic cancer. Consistent with the above findings, we also observed that vitamin D supplementation resulted in a significant absolute increase in patients with lung and prostate cancer while those with colorectal cancer recorded the lowest absolute improvement. One potential explanation on why patients with colorectal cancer showed less benefit as compared to those with lung cancer could be the more severe gastrointestinal toxicity (stomatitis and diarrhea) associated with chemotherapy regimens for colorectal cancer [36]. Severe stomatitis could have an effect on compliance or ability to take an oral supplement, while severe diarrhea could impact intake as well as absorption. This explanation seems to be consistent with the findings from a study which observed an association between chemotherapy and a significant increase in the risk of severe vitamin D deficiency in colorectal cancer patients [36]. The study hypothesized that chemotherapy administration in colorectal cancer might result in dietary modifications such as reduction or elimination of milk products as part of the management of chemotherapy- induced diarrhea. It also surmised that patients undergoing chemotherapy might not absorb dietary vitamin D well due to subclinical mucositis [36]. As a result, these patients may need higher amounts of supplementation for a longer period of time in order to achieve adequate serum 25(OH)D status. Future studies should evaluate response to supplementation in relation to different chemotherapy regimens.

The response to supplementation from suboptimal to optimal levels was greatest in patients with baseline 25(OH)D levels between 20-32 ng/ml as compared to those below 20 ng/ml. Also, vitamin D supplementation resulted in a significant absolute increase in serum 25(OH)D levels particularly in patients with lower levels at baseline. This is not surprising because patients with lower baseline levels have to cover a greater ground to convert from suboptimal to optimal levels. On the other hand, those with higher baseline levels are more likely to convert. The observation of highest absolute improvement in serum 25(OH)D in those with lowest baseline levels is also corroborated by previous research conducted in different patient populations including elderly, premenopausal and healthy individuals which found that participants with lower baseline serum 25(OH)D concentrations had a stronger serum 25(OH)D response to supplementation [40-44]. It has been hypothesized that hydroxylation of vitamin D3 to 25(OH)D is likely a saturable process, causing an attenuated response to supplementation in individuals with higher baseline serum 25(OH)D concentrations [45].

In order to put our study in context, we review here 3 studies in breast cancer that have evaluated the impact of vitamin D supplementation on serum 25(OH)D levels. Crew et al. examined the effects of standard-dose vitamin D supplementation on serum 25(OH)D levels in breast cancer patients. They observed that cholecalciferol 400 IU daily for 1 year raised serum 25(OH)D levels only modestly, by less than 3 ng/mL in only a small percentage of premenopausal women (< 15%). Although the RDA of vitamin D in premenopausal women is only 200 IU daily, their study suggested that a dose of 400 IU daily was inadequate in breast cancer patients, even to maintain skeletal health, and was probably too low for meaningful anticancer effects [28].

The other study conducted on breast cancer patients by Khan et al. reported the safety and efficacy of vitamin D supplementation using 50,000 IU weekly on postmenopausal women. They studied the effect of vitamin D-ss (standard supplementation) and vitamin D-HD (high dose) supplementation on serum 25(OH)D levels. According to them vitamin D-HD for 12 weeks is extremely effective in optimizing 25(OH)D levels and results in a predictable increase in 25(OH)D levels. Comparing women who received vitamin D-HD versus vitamin D-ss, the former displayed statistically significant higher values. Moreover, vitamin D-HD was safe in this population, with no cases of hypercalcemia or renal stones. Their results also suggested that 50,000 IU of vitamin D3, when given weekly to post-menopausal women starting adjuvant letrozole, resulted in clinically significant improvement in disability from joint symptoms [29].

A more recent data published by Nogues et al. reported a prevalence of 85-92% of vitamin D deficiency (defined as < 30 ng/ml) in breast cancer patients as compared to 74% reported by Crew et al. (defined as < 20 ng/ml) and 63% by Khan et al (defined as < 20 ng/ml). In this study, treatment with 16,000 IU of vitamin D every 2 weeks increased vitamin D plasma levels significantly in about 76.52% of subjects with baseline vitamin D deficiency (plasma levels < 30 ng/ml) over 3 months follow up. However, few subjects had baseline 25(OH)D levels ≥ 30 ng/ml and were prescribed the normal daily calcium and vitamin D supplements (800 IU) and their 25(OH)D levels did not increase significantly [30].

The dosage used for vitamin D supplementation in our study was 8000 IU/d, consistent with the data from Holick MF of 50,000 IU weekly [33], which led to an increase in the mean serum 25(OH)D levels from 19.1 ng/ml to 36.2 ng/ml. No safety concerns were reported. When comparing it with the vitamin D dose response in healthy individuals, the literature yielded the following results. Talwar et al. showed that supplementation with 800 IU/d vitamin D3 in postmenopausal African American women raised the mean serum 25(OH)D concentration from a baseline of 18.7+/-8.2 ng/mL to 28.5+/-8.6 ng/mL at a 3 month interval [46]. In another study, Barger-Lux et al. showed that in a relatively replete group of white subjects, 1000 IU vitamin D3/d resulted in an increase of 5.2 ng/mL from a mean of 26.8 to 32 ng/mL [45]. Likewise Heaney et al reported a dose response of 0.28 ng/mL per 1 μg/40IU oral vitamin D3 supplemented [47]. Furthermore, Aloia et al. undertook a dose-finding study in African American and white men and women with the objective of investigating an algorithm for raising 25(OH)D concentrations to between 32 and 56 ng/mL. They suggested a dose of 3800 IU for those above a 25(OH)D threshold of 22 ng/mL and a dose of 5000 IU for those below that threshold [16].

Our study has some limitations. This study, because of its retrospective nature, relies on data not primarily meant for research. As a result, we could not adjust for several potential confounding factors that could have influenced serum 25(OH)D levels. For example, we did not adjust for season of blood draw in our analyses. Therefore, increase in serum 25(OH)D levels between baseline and first follow-up could have been influenced by change in season of blood draw, especially since the time between assessments could have put a patient into a different season by the first assessment. Moreover, we did not have information on intake of vitamin D or data regarding their typical sun exposure which could have shed further light on the subjects' vitamin D status. However, our patients reside in all areas of the United States. Other variables such as race, physical activity, BMI and chemotherapy received known to have a significant relationship with 25(OH)D status were not controlled for in the analyses. Without a control group, it is less clear whether vitamin D insufficiency preceded the diagnosis of cancer or whether it was associated with the disease process. We did not collect information about compliance with vitamin D supplementation, which may be an important element to understand the response to vitamin D supplements in certain individuals. For some non-responders that were questioned about compliance, compliance was an issue, as well as the type of vitamin D supplement that was being used. On at least one occasion, a patient taking hard tablets did not respond, and subsequently responded when switched to a capsule. This raises another limitation of our study. In our study, patients were encouraged to use a capsule form of vitamin D3, however, some did choose to use tablets or liquids. The time to first follow-up was not uniform due to differences in medical treatment regimens, and there were several losses to follow-up in the study. This is because many patients decided not to get treated at our hospital after their initial evaluation. Also some patients could not return for follow up due to the advanced nature of their disease or death. A poor follow-up rate of 48.4% introduces selection bias into the study because patients available for first follow-up (n = 799) differed from those not available (n = 852) with regard to several baseline characteristics such as cancer type, stage, gender and treatment history. Since 25(OH)D assays were measured in real-time rather than batches in a central laboratory, this may introduce batch to batch variability in the assays. Most of the literature on vitamin D and cancer (including our study) is largely based upon observational data. Therefore, rigorous clinical trials are needed before we can make broad recommendations about high-dose supplementation to our patients.

There are several clinical implications of this work such as the need to monitor the vitamin D intake and serum 25(OH)D levels in patients with cancer and supplementation of vitamin D in those who are found to have suboptimal levels. Vitamin D insufficiency is prevalent in this population and should be routinely assessed, especially in breast and prostate cancer patients where the treatment for these diseases also has an impact on long-term bone health. There was a significantly lower response to supplementation in individuals with colorectal and pancreatic cancers, suggesting these individuals may need higher doses of supplementation for longer periods of time and/or may have a higher rate of noncompliance. As a result, assessing and monitoring compliance to oral supplementation is critical in colorectal and pancreatic cancer patients where oral intake and absorption may be compromised. Further studies evaluating the higher dose given just once weekly may help answer the question of whether compliance and/or absorption contributes to a less than average response in patients with colorectal cancer. Determining the effects of achieving and maintaining adequate 25(OH)D levels with supplementation on patient outcomes is also an important research avenue.

## Conclusions

We found oral vitamin D formulation of 8000 IU/d for 8 weeks to be a safe regimen to correct vitamin D insufficiency in this oncology population. The response to supplementation from suboptimal to optimal levels was greatest in patients with prostate and lung cancer as well as those with baseline levels between 20-32 ng/ml and lowest in those with colorectal and pancreatic cancer as well as those with baseline levels below 20 ng/ml. Characteristics of non-responders as well as those who take longer to respond to supplementation need to be further studied and defined. Additionally, the impact of improved serum 25(OH)D levels on patient survival and quality of life needs to be investigated. Further prospective studies are needed in this direction.

## Competing interests

The authors declare that they have no competing interests.

## Authors' contributions

PGV was the main author of the manuscript, participated in concept, design, writing and data interpretation. KT and CAL participated in concept, design, data collection and writing. DPB participated in concept, design and data interpretation. DG participated in concept, statistical analysis, data interpretation and writing. All authors read and approved the final manuscript.
